# Predicting the risk of childhood overweight and obesity at 4–5 years using population-level pregnancy and early-life healthcare data

**DOI:** 10.1186/s12916-020-01568-z

**Published:** 2020-05-11

**Authors:** Nida Ziauddeen, Sam Wilding, Paul J. Roderick, Nicholas S. Macklon, Dianna Smith, Debbie Chase, Nisreen A. Alwan

**Affiliations:** 1 School of Primary Care, Population Sciences and Medical Education, Faculty of Medicine, University of Southampton, Southampton, UK; 2 Department of Obstetrics and Gynaecology, University of Copenhagen, Zealand University Hospital, Roskilde, Denmark; 3 London Women’s Clinic, 113-115 Harley Street, London, UK; 4 Geography and Environmental Science, Faculty of Environmental and Life Sciences, University of Southampton, Southampton, UK; 5 Public Health, Southampton City Council, Southampton, UK; 6 NIHR Southampton Biomedical Research Centre, University of Southampton and University Hospital Southampton NHS Foundation Trust, Southampton, UK

**Keywords:** Pregnancy, Early life, Overweight, Obesity, Prediction

## Abstract

**Background:**

Nearly a third of children in the UK are overweight, with the prevalence in the most deprived areas more than twice that in the least deprived. The aim was to develop a risk identification model for childhood overweight/obesity applied during pregnancy and early life using routinely collected population-level healthcare data.

**Methods:**

A population-based anonymised linked cohort of maternal antenatal records (January 2003 to September 2013) and birth/early-life data for their children with linked body mass index (BMI) measurements at 4–5 years (*n* = 29,060 children) in Hampshire, UK was used. Childhood age- and sex-adjusted BMI at 4–5 years, measured between September 2007 and November 2018, using a clinical cut-off of ≥ 91st centile for overweight/obesity. Logistic regression models together with multivariable fractional polynomials were used to select model predictors and to identify transformations of continuous predictors that best predict the outcome.

**Results:**

Fifteen percent of children had a BMI ≥ 91st centile. Models were developed in stages, incorporating data collected at first antenatal booking appointment, later pregnancy/birth, and early-life predictors (1 and 2 years). The area under the curve (AUC) was lowest (0.64) for the model only incorporating maternal predictors from early pregnancy and highest for the model incorporating all factors up to weight at 2 years for predicting outcome at 4–5 years (0.83). The models were well calibrated. The prediction models identify 21% (at booking) to 24% (at ~ 2 years) of children as being at high risk of overweight or obese by the age of 4–5 years (as defined by a ≥ 20% risk score). Early pregnancy predictors included maternal BMI, smoking status, maternal age, and ethnicity. Early-life predictors included birthweight, baby’s sex, and weight at 1 or 2 years of age.

**Conclusions:**

Although predictive ability was lower for the early pregnancy models, maternal predictors remained consistent across the models; thus, high-risk groups could be identified at an early stage with more precise estimation as the child grows. A tool based on these models can be used to quantify clustering of risk for childhood obesity as early as the first trimester of pregnancy, and can strengthen the long-term preventive element of antenatal and early years care.

## Background

In 2016, 50 million girls and 74 million boys worldwide were obese [1]. Childhood obesity has adverse effects on cardiovascular structure and function, with increased lifetime risk of cardiovascular disease [2]. Overweight or obese children are over four times more likely to also have overweight or obesity at age 15 [3]. With high rates of overweight and obesity and evidence of tracking of weight status from childhood to adolescence to adulthood [4], a higher proportion of the population is being exposed to obesity for longer. Obesity contributed to 617,000 hospital admissions in England in 2016/2017, an 18% increase from the year before (2015/2016) [5]. Data from the National Child Measurement Programme (NCMP) in England showed that in 2017/2018, 22% of children aged 4 to 5 years and 34% aged 10 to 11 years were classified as overweight or obese [6]. Children living in the most deprived areas in England were twice as likely to be obese than children in the least deprived areas, and this gap has shown an increase over the last decade [6].

Intervening early to prevent childhood obesity is key to tackling the obesity epidemic [7]. Prevention is a central theme of the UK Government’s vision for the nation’s health [8–11]. Key to effective prevention, complementing population-level policy change and intervention, is identifying groups at risk for targeted support. The first ‘1000’ days, the time from conception to age 2 years, is recognised to be a critical period of development. Maternal factors and early-life factors consistently shown to be associated with risk of childhood overweight and obesity during this period include maternal pre-pregnancy overweight and obesity [12, 13], maternal smoking during pregnancy [13, 14], maternal excessive gestational weight gain [14], high birthweight [13, 14], and rapid infant weight gain in the first 2 years [13, 14]. Gestational diabetes (GDM) has also been identified as a risk factor [14]. Maternal educational attainment [15–17] and employment status [18–20] are also risk factors, but fewer studies have examined these associations. Evidence on breastfeeding and maternal postnatal depression as risk factors for childhood obesity is less consistent [14]. Research using birth cohort data from Singapore [21] and the UK [22] has shown that having a greater number of pregnancy and early-life risk factors increases the risk of childhood overweight and obesity. However, quantifying the effect of this combined risk in clinical practice to target interventions using routinely available data is less explored.

Risk prediction based on single predictive factors tends to have poor prognostic accuracy. The use of multiple predictive factors combined in a prediction model improves the prediction [23]. Better predictive information is beneficial to identify children at risk early thus providing the ability to target interventions and support at an earlier stage. A systematic review of existing prediction models of overweight and obesity in childhood found eight existing models developed using data from Greece, Finland, Germany, USA, the Netherlands, Seychelles, and two from the UK [24]. Only two of these eight models could be applied to routinely collected healthcare data in the UK. The other six models included predictors related to the father (such as paternal body mass index (BMI) or employment) or household (such as smoking in the household and income) which are not measured routinely as part of antenatal or postnatal healthcare records. Both models that could be applied to a routine dataset included the same predictors—maternal BMI, birthweight (*z*-score), early-life weight gain (*z*-score), and infant gender. However, there are other maternal and child variables that can be included to potentially enhance predictive power and do not involve extra data collection by healthcare professionals in their everyday practice, and this is what we set out to test.

We aimed to develop and internally validate prediction models of childhood overweight and obesity using antenatal, birth, and early-life data, all of which are routinely collected as part of healthcare records, utilising an objective measure of weight status at school age. This offers the unique perspective of assessing whether routinely collected data can be used to predict overweight and obesity at an early stage with reasonable accuracy, and ultimately whether such a childhood obesity risk estimation tool can be effectively applied during pregnancy and early life to help healthcare professionals target extra support towards high-risk families.

## Methods

SLOPE (Studying Lifecourse Obesity PrEdictors) is a population-based anonymised linked cohort of maternal antenatal and birth records and child health records for all births registered at University Hospital Southampton (UHS), in the South of England between 2003 and 2018. UHS provides maternity care to residents in the city of Southampton and the surrounding areas of Hampshire. Child healthcare for the same area is provided by two community National Health Service (NHS) Trusts: Solent and Southern Health. Thus, the antenatal and birth records (*n* = 83,481) were then linked to child health data from these two community NHS Trusts (*n* = 74,770, 90% linked). Only singleton pregnancies with feasible gestational age, maternal weight, and maternal height measurements were included in this analysis. All the variables described below are routinely collected for pregnant women and children receiving healthcare in the study region.

### Outcome measurement

As part of the NCMP, the height and weight of children in all state-maintained schools in England are measured by school nurses at year *R* (4–5 years) and year 6 (10–11 years) [25]. In the UK, children start school in the September after their fourth birthday and they can be measured at any point during the school year. For the purposes of this study, this was defined as the first measurement of weight and height on the same day between the ages of 4 and 6 years. Thus, all children with a valid weight and height measurement at reception year constituted the sample for outcome (*n* = 30,958). BMI was then calculated as weight/(height)^2^ and converted to age- and sex-adjusted BMI *z*-scores according to the UK 1990 growth reference charts [26]. A *z*-score of + 1.33 equates to the 91st percentile, and this was used to specify the outcome of childhood overweight and obesity used in the prediction models. This cut-off was chosen as it is the most relevant to healthcare professionals in the UK, given it is the one used by national guidance on the clinical management of childhood overweight [27, 28], and has been used in another UK-based prediction model [29].

### Candidate predictors

The prediction model was developed in stages, incorporating data collected at the booking appointment, birth, and early life (1 and 2 years of age). Thus, model predictors were identified at each of these stages. For later time points (birth and early life), the candidate predictors are in addition to the candidate predictors from the earlier stage.

#### First antenatal (booking) appointment

The first antenatal booking appointment is recommended to ideally take place by the 10th week of gestation, according to the National Institute for Health and Care Excellence (NICE) Guidelines [30]. Maternal age (in years) was calculated from date of birth in the clinical electronic records. Maternal BMI was calculated using weight in kilogrammes measured at the booking appointment by the midwife and self-reported height. Smoking was self-reported as current smoker, ex-smoker, or non-smoker. Highest maternal educational qualification was categorised as secondary (GCSE) and under, college (A levels), and university degree or above. Self-reported ethnicity was recorded under 16 categories and condensed to White, Mixed, Asian, Black/African/Caribbean, and Other. Employment status was categorised as employed, unemployed, and in education. Intake of folic acid supplements was categorised as taking before becoming pregnant, started taking once pregnant, and not taking supplement. Maternal first language English, history of stillbirth/miscarriage, previous caesarean section, maternal disability status, maternal substance use, and partnership status were categorised as yes or no. Infertility treatment was categorised as no, yes (hormonal only, in vitro fertilisation, gamete intrafallopian transfer, and other surgical), and investigations only. Maternal history, obstetric history, and family history were asked as separate questions such as ‘do you have existing medical conditions’ and recorded if any existing conditions were reported to each of the three questions. Parity was recorded as the number of previous live births reported and condensed to 0, 1, 2, and ≥ 3 for this analysis. Maternal diet was recorded as no special diet, pescatarian, vegetarian, vegan, and other.

#### Birth

Birthweight (grammes) was measured by healthcare professionals at birth. Gestational age was based on a dating ultrasound scan which takes place between 10 weeks and 13 weeks 6 days gestation [30]. Mode of birth was categorised as vaginal and caesarean. In this population, an oral glucose tolerance test was used for screening for GDM in women with one or more risk factors (BMI > 30 kg/m^2^; GDM in previous pregnancy; previous baby weighing ≥ 4.5 kg; diabetes in parents or siblings and of Asian, African-Caribbean, or Middle Eastern ethnicity) [31]. GDM diagnosis was then reported in the database. Pre-eclampsia was reported as yes or no.

#### Early life

Breastfeeding status was reported at hospital discharge and during early life. The recording during early life was done differently by the two community NHS Trusts. One used NHS Read codes and thus was recorded at 10 days, 2 weeks, 6 weeks, 4 months, and 9 months. At each point, this was recorded as breastfed, bottle-fed, or breast and bottle-fed. Breastfeeding could be recorded at any or all of the time points specified by the Read codes. The other Trust recorded breastfeeding at 56 days (8 weeks) as yes or no, so there was no information on whether this was exclusive or partial breastfeeding. The 10-day and 2-week categories were combined into one as there were very few instances of the 2-week category recorded and the two categories are only 4 days apart. Using all the information available, a breastfeeding variable was derived with categories of no breastfeeding, minimum 10 days, minimum 6 weeks, minimum 8 weeks, minimum 4 months, and minimum 9 months. Minimum duration was chosen as there was no information how long breastfeeding was continued for beyond the point of the last record.

Early-life weight was calculated at two ages—1 year and 2 years. To maximise the number of records and accounting for the routine development checks offered within the NHS where children are measured (9 to 12 months and 2 to 2.5 years), weight measured between 9 and 13 months was used as the 1-year weight. Similarly, weight measured between 23 and 30 months was used as the 2-year weight. In the 2-year models, we have not included the 1-year weight measured to maximise sample size.

In sensitivity analysis, we assessed whether the inclusion of area-level characteristics improves the discrimination of the birth prediction model. Geographical area at birth was characterised for children living in the Southampton area [32]. The unit of analysis was Lower layer Super Output Area (LSOA) boundaries in order to preserve anonymity. These areas have an average population of around 1500 and cover an average area of 4 km^2^. Area characteristics for these LSOAs were collated and included the 2015 Index of Multiple Deprivation (IMD), household median price quintile, measures of air pollution (PM_2.5_, PM_10_, NOx), Income Support claimant rate, social renting households, supermarket density, unhealthy food index, greenspace, spaces for social interaction, and walkability. Further information on how the area-level data were sourced, collated, and derived is available on the project website: https://www.southampton.ac.uk/slope/data/area-data.page.

### Statistical analysis

All analysis was performed using Stata 15 [33]. Clustering by mother was adjusted for by including a clustering indicator in the model as some women had more than one pregnancy in the dataset. Both complete case and multiple imputed analysis was carried out. Multiple imputation by chained equations (MICE) was carried out using truncated regression for continuous variables and predictive mean matching for categorical variables. The sample was limited to those with the outcome of interest, and only missing predictor values were imputed. The percentage of missing data was low for the antenatal and birth data (5%), but there was a high percentage of missing data during early life for all variables at this stage (70%) and so we carried out 70 imputations of the sample generating 70 imputed datasets (with 10 iterations per imputed dataset). This was based on the recommendation that the number of imputations equals the percentage of missing data in the dataset [34]. The estimated regression parameters (coefficients and variances) were combined over the imputed datasets using Rubin’s rules [34].

Stepwise backward elimination was used to select variables to be included in the model [35]. This automatic selection procedure starts with the full model (including all candidate predictor variables) and sequentially removes variables based on a series of hypothesis tests. Automatic selection procedures are data driven and make decisions regarding inclusion/exclusion of variables based on hypothesis tests with a pre-specified significance level for inclusion/exclusion. In backward elimination, variables are removed sequentially if the *p* value for a variable exceeds the specified significance level which was set at 0.157 for this analysis which was chosen conservatively to reduce the risk of overfitting. This is equivalent to the Akaike information criterion (AIC) [36].

As the outcome was binary, the models were developed using logistic regression. All continuous variables were retained as continuous to avoid loss of information. Fractional polynomials were used to investigate non-linear relationships between continuous candidate predictors and the outcome. The best transformation for each continuous predictor was identified through backward elimination with the selection of a fractional polynomial function by starting with the most-complex permitted fractional polynomial and attempt to simplify the model by reducing the degree. This was then used when fitting the models.

Events per variable (EPV) is used to ensure that the sample size is large enough to avoid issues related to precision and overfitting especially when using automatic selection procedures. A rule of thumb from simulation studies is that there should be a minimum of 10 events per variable [37, 38]. Considering this in model development, there were sufficient cases of the outcomes to develop a prediction model using booking and birth factors. However, there were insufficient cases of outcome during early life in the complete case analysis so it was decided to include fewer candidate predictors at the model development stage in the early-life models. Predictors included were guided by the literature (maternal age, maternal BMI, ethnicity, smoking status, educational attainment, birthweight, child sex) and those that remained in the booking and birth models.

Bootstrapping (1000 repetitions) was chosen as the method for internal validation as this method provides stable estimates with low bias [39]. It also provides an estimate of the expected optimism which can be used to weight down the model parameter estimates. Internal validation was only carried out in complete case models. This was because the complexities involved in model development (combination of variable selection, fractional polynomials, and multiple imputation) in the multiple imputation models meant that the steps involved could not replayed. Instead, apparent model performance was assessed in the multiple imputed models and compared to the complete case models.

To test if the prediction models at birth could be improved with the inclusion of area-level characteristics at birth, we first included IMD as an additional candidate predictor. We then excluded education as a candidate predictor as population-level educational attainment is included in the IMD. All individual and area-level predictors as outlined above were then considered for inclusion in the final birth model.

### Model performance and shrinkage

Model performance was assessed using discrimination and calibration*.* Discrimination is a measure of how well the model differentiates between individuals [37]. The area under the curve (AUC) was used to summarise the overall discriminatory ability of the models. The AUC was classified as 0.6–0.7 poor, 0.7–0.8 fair, 0.8–0.9 good, and 0.9–1.0 excellent.

Calibration measures how well the predicted outcome of the model agrees with the observed outcome on average. The predicted probability (*x*-axis) is plotted against the observed outcome proportion (*y*-axis) for each risk group. The slope of a line fitted through the points on the graph is the calibration slope and has been calculated for the models. Calibration slope would be one in a perfectly calibrated model. A slope of less than one or greater than one indicates over- and under-prediction, respectively [40]. The recommendation of overlaying calibration curves from each imputed dataset in the calibration plots was followed [41].

Prediction models tend to be optimistic in the development data as a result of overfitting, and use of a newly developed model in independent data tends to lead to worse predictions. Heuristic shrinkage factors were calculated for each model to estimate the extent of overfitting present in the developed models [42]. The heuristic shrinkage factor is calculated as:

$$ \left(\mathrm{model}\ {\chi}^2-\mathrm{df}\right)/\mathrm{model}\ {\chi}^2 $$

where model *χ*^2^ is the model likelihood ratio and df is the degrees of freedom in the fitted model. A shrinkage factor of 1 implies no shrinkage.

The regression coefficients from the models were multiplied by the shrinkage factor to adjust the models for optimism. A logistic model was then fitted for the outcome to estimate the shrinkage of the intercept by including the linear predictor calculated using the shrunken coefficients as the only independent variable and constraining its coefficient to one.

Sensitivity, specificity, positive predictive value (PPV), and negative predictive value (NPV) were calculated at multiple risk score cut-off points as no standard criteria for identifying a risk threshold exist for the prediction of childhood obesity. As the models have been designed in a sequential manner and risk can be calculated at each of these stages, the accumulation of risk for individuals over time if the model is applied at each of these stages was calculated.

### Calculating risk score

The log-odds (*Y*) can be calculated using the regression equation as follows:

$$ Y=\mathrm{constant}+\left[{\mathrm{estimate}}_1\times {\mathrm{predictor}}_1\right]+\left[{\mathrm{estimate}}_2\times {\mathrm{predictor}}_2\right]+\dots +{\mathrm{estimate}}_n\times {\mathrm{predictor}}_n\Big] $$

The log-odds (*Y*) is then converted into probability (*P*) as follows:

$$ P=1/\left[1+\exp \left(-Y\right)\right] $$

where *P* is the probability of developing the outcome and *Y* is the log-odds estimated using the model.

## Results

**Fig. 1:**
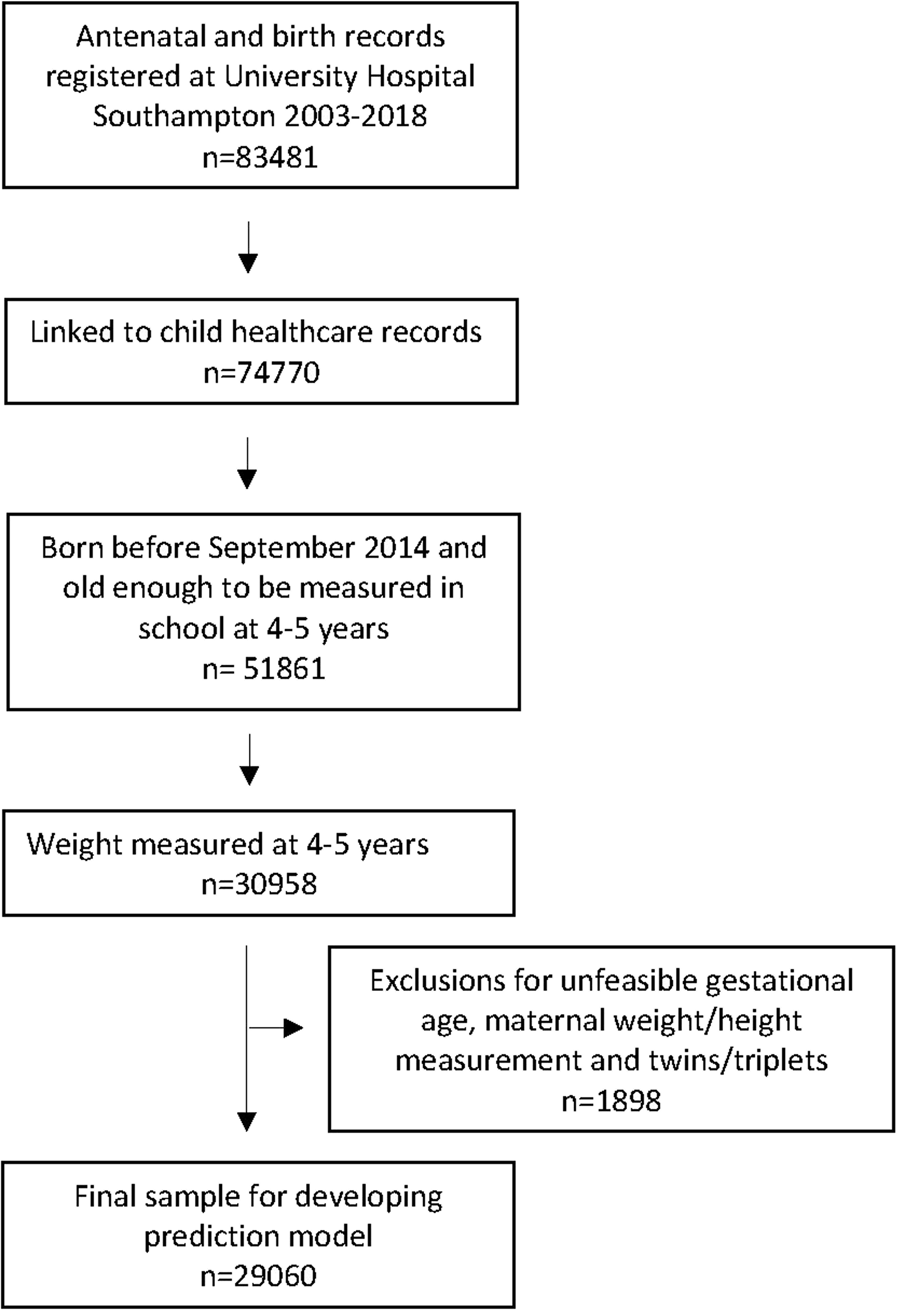
Flow diagram showing the eligible sample

Of the 51,861 children who were old enough to be in school (4–5 years) based on the maternal dataset, outcome data were included in the linked dataset for 30,958 children (60%). This reduced to 29,060 children after exclusions for unfeasible gestational age, maternal weight and maternal height measurements, and multiple births (twins/triplets) (Fig. 1). Of these, 4311 children (14.8%) were overweight/obese (≥ 91st centile for age and sex). Baseline characteristics are summarised in Table 1. Maternal age at booking was 28.4 years (standard deviation (SD) 5.9). Mean maternal BMI at booking was 25.5 kg/m^2^ (SD 5.5). Over 50% of women reported being ex- (33.6%) or current (17.4%) smokers. A quarter of the women had a university degree or a higher qualification, and over two thirds were employed at the first antenatal (booking) appointment. Eight percent of mothers reported being a lone parent at the booking appointment. Nearly half the mothers reported no breastfeeding.

**Table 1:** Summary of baseline characters (candidate predictors) and outcome for the SLOPE sample using the multiple imputed data

Stage recorded	Variable	Mean ± SD
	*N*	29,060
Booking appointment	Maternal age at booking, years	28.4 ± 5.9
Booking appointment	Gestation at booking, days	80 ± 19
Booking appointment	Maternal BMI at booking, kg/m^2^	25.5 ± 5.5
Birth	Birthweight, kg	3.4 ± 0.6
Birth	Gestation at birth, days	279 ± 13
Early life (~ 1 year)	Weight at 1 year, kg	9.4 ± 1.2
Early life (~ 1 year)	Weight at 2 years, kg	13.0 ± 1.6
		%, 95% CI
Booking appointment	Maternal smoking status at booking	
	- Never smoked	49.0 (48.4 to 49.5)
	- Ex-smoker	33.6 (33.1 to 34.2)
	- Current smoker	17.4 (17.0 to 17.9)
Booking appointment	Maternal highest educational attainment	
	- University degree or above	25.5 (25.0 to 26.0)
	- College (A levels)	40.9 (40.4 to 41.5)
	- Secondary school or below	33.6 (33.1 to 34.1)
Booking appointment	Maternal employment status at booking	
	- Employed	68.9 (68.4 to 69.5)
	- Unemployed	28.8 (28.3 to 29.4)
	- Student or in training	2.2 (2.1 to 2.4)
Booking appointment	Maternal ethnicity	
	- White	90.4 (90.0 to 90.7)
	- Mixed	1.1 (1.0 to 1.2)
	- Asian	6.1 (5.8 to 6.3)
	- Black/African/Caribbean	1.5 (1.3 to 1.6)
	- Other	1.0 (0.9 to 1.1)
Booking appointment	Intake of folic acid supplements	
	- Taking prior to pregnancy	31.0 (30.4 to 31.5)
	- Started taking once pregnant	58.3 (57.7 to 58.8)
	- Not taking supplement at booking	10.8 (10.4 to 11.1)
Booking appointment	Maternal first language English	97.1 (96.9 to 97.3)
Booking appointment	Partnership status at booking	91.7 (91.4 to 92.0)
Booking appointment	Infertility treatment	
	- No	92.5 (92.2 to 92.8)
	- Yes	4.2 (4.0 to 4.4)
	- Investigations but no treatment	3.3 (3.1 to 3.5)
Booking appointment	History of mental health	20.5 (20.1 to 21.0)
Booking appointment	Previous stillbirth	0.8 (0.7 to 0.9)
Booking appointment	Parity at booking	
	- 0	45.0 (44.5 to 45.6)
	- 1	35.2 (34.6 to 35.7)
	- 2	13.0 (12.6 to 13.4)
	- ≥ 3	6.8 (6.5 to 7.1)
Booking appointment	Previous caesarean section	
	- 0	87.9 (87.5 to 88.2)
	- 1	10.1 (9.7 to 10.4)
	- 2	2.0 (1.9 to 2.2)
Booking appointment	Maternal diet	
	- No special diet	93.3 (93.0 to 93.6)
	- Pescatarian	2.3 (2.2 to 2.5)
	- Vegetarian	2.2 (2.1 to 2.4)
	- Vegan	0.1 (0.1 to 0.1)
	- Other	2.1 (1.9 to 2.2)
Booking appointment	Maternal disability status	1.0 (0.9 to 1.1)
Booking appointment	Maternal substance use	0.1 (0.1 to 0.2)
Booking appointment	Obstetric history of GDM	0.9 (0.7 to 1.0)
Booking appointment	Obstetric history of preeclampsia	0.2 (0.1 to 0.2)
Booking appointment	Family history of diabetes	14.2 (13.8 to 14.6)
Booking appointment	Family history of hypertensive disorder	31.6 (31.0 to 32.1)
Booking appointment	Family history of mental health conditions	16.1 (15.7 to 16.6)
Birth	Delivery method	
	- Vaginal	77.8 (77.3 to 78.2)
	- Caesarean section	22.2 (21.8 to 22.7)
Birth	Gestational diabetes in current pregnancy	2.0 (1.9 to 2.2)
Birth	Preeclampsia in current pregnancy	0.5 (0.4 to 0.6)
Birth	Child sex	
	- Male	51.2 (50.6 to 51.8)
	- Female	48.8 (48.2 to 49.4)
Early life	Duration of breastfeeding	
	- No breastfeeding	48.2 (47.4 to 49.0)
	- Minimum 10 days	19.6 (19.0 to 20.2)
	- Minimum 6 weeks	21.8 (21.1 to 22.5)
	- Minimum 8 weeks	9.5 (9.1 to 10.0)
	- Minimum 4 months	0.3 (0.2 to 0.4)
	- 9 months	0.6 (0.4 to 0.7)
4–5 years	Childhood overweight or obesity (≥ 91st centile)	14.8 (14.4 to 15.2)

The prediction models for the risk of childhood overweight and obesity are presented in Table 2 and Additional file 1: Table S1. Eight predictors were retained in the final model at booking: maternal age, BMI, smoking status, ethnicity, intake of folic acid supplements, first language, partnership status, and parity. For the outcome at birth, the same predictors were retained in the final model with the addition of maternal educational attainment, birthweight, and gestational age at birth. At early life, all maternal predictors with the exception of mother’s first language and parity were retained. Child predictors included birthweight, sex, and weight at ~ 1 or ~ 2 years, respectively. Gestational age at birth remained a predictor at ~ 1 year but not at ~ 2 years. Transformations were identified for maternal age, maternal BMI, and birthweight.

**Table 2:** Intercept and regression coefficients of the prediction models for overweight and obesity (≥ 91st centile) in children aged 4–5 years before and after shrinkage

Predictors	Booking		Birth		Early life (~ 1 year)		Early life (~ 2 years)	
	Coefficient	Shrunken coefficient	Coefficient	Shrunken coefficient	Coefficient	Shrunken coefficient	Coefficient	Shrunken coefficient
Intercept	0.877	0.845	2.215	2.169	− 5.186	− 5.160	− 10.510	− 10.466
Maternal age at booking, years	1.394	1.377	1.114	1.101	− 0.006	− 0.006		
Maternal BMI at booking, kg/m^2^	− 7.061	− 6.971	− 6.371	− 6.295	− 6.733	− 6.686	− 6.687	− 6.656
Maternal smoking status at booking								
Never smoked	Ref		Ref		Ref		Ref	
Ex-smoker	0.099	0.098	0.080	0.079	0.047	0.047	0.044	0.044
Current smoker	0.436	0.431	0.583	0.576	0.532	0.528	0.536	0.534
Maternal educational attainment								
University or above			Ref		Ref		Ref	
College			0.088	0.087	0.130	0.129	0.116	0.115
Secondary or lower			0.103	0.102	0.190	0.188	0.174	0.173
Maternal ethnicity								
White	Ref		Ref		Ref		Ref	
Mixed	0.020	0.020	0.105	0.103	0.098	0.098	0.019	0.019
Asian	0.274	0.271	0.444	0.439	0.589	0.585	0.402	0.400
Black/African/Caribbean	0.655	0.647	0.778	0.769	0.771	0.766	0.511	0.509
Other	0.084	0.083	0.124	0.122	0.235	0.234	− 0.073	− 0.073
Maternal intake of folic acid supplements								
Taking prior to pregnancy	Ref		Ref		Ref		Ref	
Started taking once pregnant	0.094	0.093	0.120	0.118	0.156	0.155	0.155	0.154
Not taking supplement	0.053	0.053	0.084	0.083	0.160	0.158	0.159	0.159
Maternal first language English								
No	Ref		Ref					
Yes	− 0.319	− 0.315	− 0.285	− 0.282				
Partnership status at booking								
Partnered	Ref		Ref		Ref		Ref	
Single	0.182	0.179	0.193	0.190	0.196	0.195	0.176	0.175
Parity at booking								
0	Ref		Ref					
1	0.017	0.016	− 0.108	− 0.106				
2	0.093	0.092	− 0.057	− 0.056				
3	0.173	0.171	0.019	0.018				
Birthweight, kg			0.107	0.106	0.129	0.128	− 0.114	− 0.113
Gestational age at birth, days			− 0.011	− 0.011	− 0.008	− 0.008		
Infant gender								
Male					Ref		Ref	
Female					0.426	0.423	0.366	0.364
Infant weight, kg					0.753	0.748	0.825	0.821
Transformations								
Maternal age at booking	(Maternal age/10)^− 2^		(Maternal age/10)^− 2^					
Maternal BMI at booking	(Maternal BMI/10)^− 1^		(Maternal BMI/10)^− 1^		(Maternal BMI/10)^− 1^		(Maternal BMI/10)^− 1^	
Birthweight			Birthweight^2^					
Discrimination and calibration								
AUC	0.66 0.65 to 0.67		0.69 0.68 to 0.70		0.78 0.77 to 0.79		0.83 0.82 to 0.84	
Calibration slope (standard error)	0.98 (0.03)		0.98 (0.03)		0.99 (0.01)		0.99 (0.01)	

Only predictors significant at *p* < 0.157 were included in the prediction models. Categorical variables with at least one significant category have been included

Predictors retained in the model in the complete case and multiple imputation were the same for the booking and birth models (Table 3). However, there were some differences in the predictors retained in the early-life models. Maternal age at booking and gestational age at birth were retained in the early-life model at ~ 1 year in the multiple imputed but not in the complete case model. Maternal ethnicity and intake of folic acid supplements were retained in all the multiple imputed models but not in the complete case early-life model at ~ 2 years. Birthweight was retained in the complete case birth model but was additionally included in both multiple imputed early-life models.

**Table 3:** Predictors included (+) in the final complete case and multiple imputed models

Predictors	Booking		Birth		Early life (~ 1 year)		Early life (~ 2 years)	
	Complete case model	Multiple imputation model	Complete case model	Multiple imputation model	Complete case model	Multiple imputation model	Complete case model	Multiple imputation model
Maternal age at booking	+	+	+	+		+		
Maternal BMI at booking	+	+	+	+	+	+	+	+
Maternal smoking status at booking	+	+	+	+	+	+	+	+
Maternal educational attainment			+	+	+	+	+	+
Maternal ethnicity	+	+	+	+	+	+		+
Maternal intake of folic acid supplements	+	+	+	+	+	+		+
Maternal first language English	+	+	+	+				
Partnership status at booking	+	+	+	+		+	+	+
Parity at booking	+	+	+	+				
Birthweight			+	+		+		+
Gestational age at birth			+	+		+		
Child sex					+	+	+	+
Child weight					+	+	+	+
Discrimination and calibration								
AUC	0.66 0.65 to 0.67	0.66 0.65 to 0.67	0.69 0.68 to 0.70	0.69 0.68 to 0.70	0.78 0.77 to 0.80	0.78 0.77 to 0.79	0.83 0.82 to 0.84	0.83 0.82 to 0.84
Calibration slope (standard error)	1.00 (0.03)	0.98 (0.03)	1.00 (0.03)	0.98 (0.03)	1.00 (0.04)	0.99 (0.01)	1.00 (0.04)	0.99 (0.01)

Discrimination (AUC) improved across the stages identified for model development from poor (0.66) at booking appointment to good (0.83) at early life (~ 2 years). AUCs were the same in the multiple imputed and complete case models after internal validation (Table 3). Sensitivity analysis including area-level predictors did not improve the model discrimination (Additional file 1: Table S2).

**Fig. 2:**
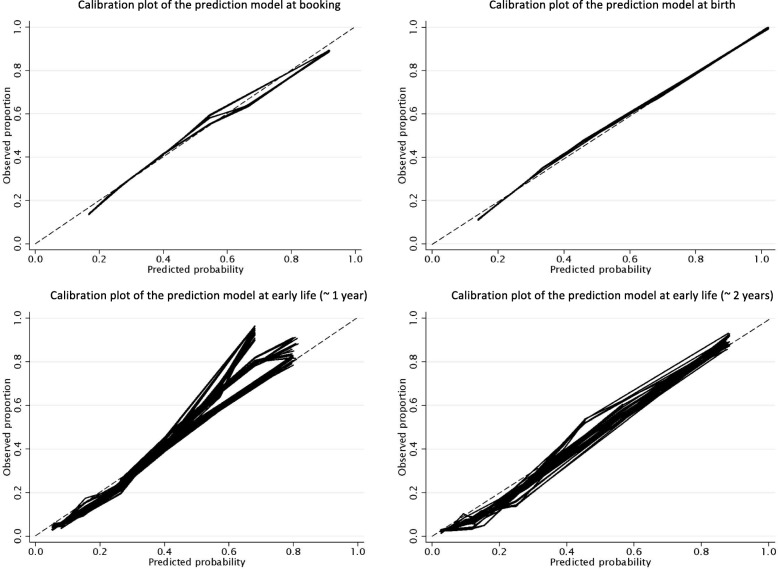
**a**–**d** Calibration plot of the prediction model at booking, birth, early life (~ 1 year), and early life (~ 2 years)

Calibration plots overlaying the results of the analysis of the imputed datasets for the model stages are presented in Fig. 2a–d. The calibrations across all models were consistently strong as evidenced by the calibration slope and the gradient. There was more variation across the imputed models in the early-life models; however, this is the stage with the highest percentage of missing data, and thus, more variation across the datasets is to be expected. The estimated shrinkage factor was 0.99 for all models suggesting that only a small percentage of the model fit was noise. The shrunken coefficients and intercepts are presented in Table 2.

The percentage of children identified as at risk of childhood overweight/obesity, sensitivity, specificity, PPV, and NPV for different risk score cut-offs are presented in Additional file 1: Table S3. As there is no agreed cut-off in the literature on what constitutes ‘high risk’ of future childhood overweight or obesity, a 20% risk threshold was used as deemed the most appropriate by the parameters reported in Additional file 1: Table S3, local stakeholder consultation and previous prediction models utilised as risk scores in routine UK healthcare [43]. For example, using a 20% risk cut-off in the early-life model at ~ 2 years identifies 24.1% of children as at risk, with a sensitivity of 65.5%, specificity of 83.1%, PPV of 40.3%, and NPV of 93.3%. The sensitivity, specificity, PPV, and NPV at each risk threshold cut-off improve across the assessment time points from pregnancy booked to 2 years of age. PPV increases and NPV decreases as the risk threshold increases, for example, for the booking model at year *R*, PPV of 18.2% and NPV of 92.7% at risk threshold of 10%, PPV of 25.9% and NPV of 88.2% at risk threshold of 20%, and PPV of 35.3% and NPV of 86.3% at risk threshold of 30%.

**Fig. 3:**
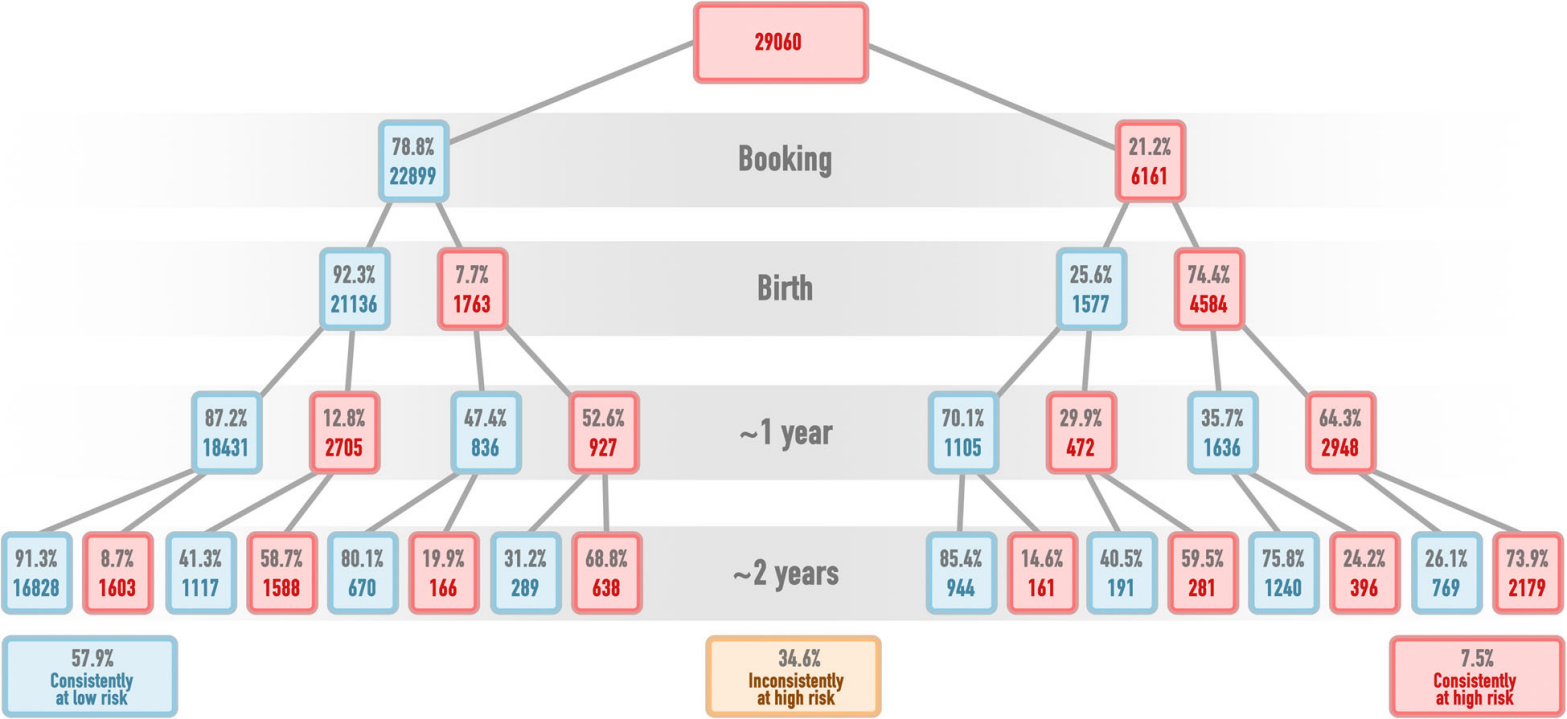
The categorisation of children as high risk (red) or low risk (blue) if the prediction model is applied at each stage using the risk threshold of 20%

Figure 3 shows the categorisation of children as high risk (≥ 20%) or not if the model is applied at each time point. Based on this, 57.9% of the sample is consistently not identified at risk from booking to 2 years of age, and 7.5% is consistently identified at risk. The remaining 34.6% are identified at risk at one or two time points but not consistently.

## Discussion

Our analysis shows that it is possible to predict childhood overweight and obesity using routine linked healthcare data collected during pregnancy and early life with reasonable accuracy. We have developed and internally validated prediction models at multiple time points starting from early pregnancy to 2 years following birth. These could be used to identify high-risk groups at each of these stages for provision of additional support/early intervention. Although the model at 2 years had better discrimination (AUC 0.83) than the models at booking (AUC 0.66), the maternal predictors remain fairly consistent across models, and thus, high-risk groups could be identified as early as the first antenatal appointment with more precise risk estimation as the child grows.

NICE guidelines in the UK recommend the use of age- and sex-adjusted BMI as a practical estimate of adiposity in children and young people to identify overweight and obesity [27]. However, the UK has different centile cut-offs for population monitoring (85th for overweight/obese and 95th for obese) and clinical diagnosis (91st for overweight and 98th for obesity) [27, 28]. Clinical cut-offs for children are used to classify children in the parental feedback letter sent as part of the NCMP. This may be because NICE guidelines have recommendations on follow-up for children who exceed this cut-off; however, this means that parents of children with BMI between the 85th (the population cut-off for overweight) and 90th percentile are being informed that their child is normal weight. The scientific rationale for the UK cut-offs is not obvious and appears to be a historical precedent selected pragmatically at the time. The 85th and 95th centiles in the UK were selected as the exact values to estimate population prevalence whereas the 91st and 98th correspond to major centile lines on the UK growth charts [28]. Although we use the clinical cut-off point for overweight/obesity in our analysis here, we have also performed the prediction models using the 85th centile cut-off and found that the included parameters are broadly the same.

Established childhood obesity risk factors that have been included in the prediction equations include maternal BMI, ethnicity, smoking status, parity, birthweight, and infant weight during early life. English as maternal first language, partnership status, and intake of folic acid supplements were consistent predictors in our models, although the evidence supporting their classification as risk factors is less strong. Offspring of women who reported English as their first language were at lower risk of childhood overweight/obesity compared to offspring of women who reported English as not their first language. This could be indicative of inequalities in healthcare access and utilisation. Lone mothers are at higher risk of poverty [44] and ill health [45] which could lead to increased levels of stress or anxiety. Folic acid supplementation could be a proxy for a variety of factors such as unplanned pregnancy, low health literacy, maternal nutrient intake status, and income. Folic acid supplements are purchased over the counter and thus could also be related to income and affordability. A systematic review found that intake of folic acid supplements from preconception reduced the risk of small-for-gestational age births [46] which is a risk factor for overweight and obesity if the child then exhibits catch-up growth in early life.

Breastfeeding was not retained as a predictor, but the evidence on the association between breastfeeding and childhood overweight and obesity is conflicting [14], and only three out of eight prediction model studies considered breastfeeding as a predictor and it was included in two models [24]. Another factor not considered in our models is gestational weight gain, which is not routinely collected in the UK. However, the additional effect of gestational weight gain on childhood obesity is small on top of the effect of pre-pregnancy weight [47–49].

The development of prediction models in a large population-based sample is a key strength of this analysis which enhances the generalisability. This is a relatively large population-based cohort of women from all socioeconomic and ethnic backgrounds resident in Southampton and surrounding areas of Hampshire and thus representative of the local population. Although Southampton is more deprived than average with the situation having worsened between 2010 and 2015 [50], about half of the women included in this analysis reside in the rest of Hampshire (the region where Southampton is situated), which is less deprived. We used robust statistical methods to develop the models (retained continuous variables as continuous, investigated variable transformations using multivariable fractional polynomials, and corrected for optimism by calculating model shrinkage) and to assess the performance of the models where possible.

There was a low percentage of missing data in the antenatal care and birth. Both early-life time points (1 and 2 years) considered in this analysis align with two of the five NHS child health and development reviews (9–12 months and 2–2.5 years) at which weight is measured. However, a high percentage of missing weight data was observed during early life (70%). Only 13.7% of children in the 2-year model also had a recorded weight at around 1 year, which prevented the inclusion of this variable at that stage. The reasons could potentially be that the measurements were entered in free text boxes or appointments did not take place within the designated period. Multiple imputation of missing data was carried out which generally enables more robust analyses but requires some caution in interpretation due to the number of imputations required. Additionally, outcome data was not available for a high proportion of children who were old enough to be in school. Factors contributing to this potentially include changes in recording practices, a child had moved and was no longer under the care of the community trust, was not attending state school, or the child NHS number (required for linkage) was not recorded with the measurement. However, the prevalence of overweight and obesity in the modelled sample was similar to the national prevalence (~ 22% using the population monitoring cut-off of 85th percentile).

This study has identified key stages for risk prediction based on routine care in the UK. No other prediction models have considered prediction as early as first trimester of pregnancy. No single risk factor was included in all eight existing prediction models identified in a systematic review of childhood obesity prediction models [24], but maternal pre-pregnancy BMI, child sex, and birthweight were the most commonly included predictors. These three predictors were retained in our models at all stages (maternal BMI) or at the appropriate stages (birthweight and child sex).

Our prediction equations have been developed using routine linked data, and so all the predictors are maternal or child factors unlike prediction models developed using birth cohort data which have incorporated paternal or family data such as paternal BMI [51] or family income [52]. The use of routine data in the development of these prediction equations means that these can be readily implemented, unlike prediction models developed using research birth cohort data incorporating data not routinely collected. For example, paternal BMI [51] could be challenging to collect in a systematic way as not all fathers attend booking appointments and missing data would be non-random. Additionally, the application of the risk prediction tool could lead to better data recording, for instance of breastfeeding status, exclusivity, and duration.

Both modifiable and non-modifiable predictors have been identified for inclusion in the models. Although these are predictors, the relationship with the outcome is not necessarily causal, and thus, interventions do not have to act on factors identified by the model. Key modifiable maternal predictors (maternal BMI, smoking status, and intake of folic acid supplements) remained consistent predictors across the stages. Identifying these high-risk families and intervening early could modify long-term risk for both mother and child as well as subsequent children particularly if identified at first pregnancy. The interpregnancy period provides a key opportunity for intervention in high-risk families for subsequent pregnancies.

The next step, following external validation of the models, is to test the feasibility, acceptability, and usability of a Childhood Obesity Risk Estimation Tool by health visitors. On public involvement, mothers have expressed interest in early identification of risk with support and advice to help modify risk. Thus, as part of the feasibility study, we are testing the use of this tool as an aid to health visitors to guide delivery of an intervention on the healthy weight pathway, rather than a screening test. Our practitioner consultation work suggests that health professionals would like an ‘objective’ way to stratify risk rather than individualised clinical judgement, as this feels subjective and can make the conversation with the family more sensitive. The risk estimation tool is envisaged to enable the provision of obesity prevention intervention at an early stage before the child is overweight or obese, to provide a prompt for the health professional to introduce this topic and to help target extra support in resource-limited settings. Although, the application of the tool may increase anxiety among parents, which will be explored as part of the feasibility study, the intervention is unlikely to produce significant unintended harm as it would not be a clinical or medicinal intervention.

We need to identify a risk threshold above which children would be considered at risk for the practical implementation of the risk estimation tool. As a definitive method for doing this could not be identified from the literature, we were guided by the sensitivity, specificity, PPV, and NPV as well as the number of individuals identified as high risk based on this threshold. For example, for outcome at year *R*, the specificity and sensitivity are comparable at a risk threshold of 15% but this identifies around 40% of the sample at risk whereas the prevalence of the outcome is 14.8%. A risk threshold of 20% would identify around 20% of the sample at risk with higher specificity but lower sensitivity and slight increase in PPV. Sensitivity and specificity are improved in the later stages (birth and early life) compared to booking.

The PPV for our 2-year model at 20% risk threshold is 40%, and the NPV is 93%. This means that a significant proportion of children identified at risk will not become overweight or obese. However, the high NPV provides confidence that very few children identified as low risk will become overweight or obese and therefore miss out on a targeted intervention. A previous childhood obesity prediction model using birth cohort data [51], which was then used to develop a prediction and intervention tool [53], had a lower PPV than ours (37%). Provided we examine the population impact and cost-effectiveness of using a risk estimation tool based on routinely collected data as a decision strategy, targeting obesity prevention interventions, which would in an ideal work be universally available if resources were not limited, is unlikely to produce harms. The potential harms of a behavioural, environmental, or social support complex intervention to tackle obesity are likely to be low compared to a clinical intervention.

Children move between low and high risk between the stages (between pregnancy and 2 years of age), but a high proportion remain consistently at high risk (7.5%). To implement this prediction in practice and intervene early, an intervention will need to take this fluctuation in risk into consideration. Withdrawing intervention or support from individuals who have previously been deemed high risk but are now low risk can have negative consequences on their risk at the next stage. However, it may not be feasible to maintain an intensive intervention during all these. Thus, interventions may need to be administered in stages or have flexible layers in terms of intensity.

## Conclusions

Most maternal predictors of childhood overweight and obesity at primary school entry remained consistent across models starting from early pregnancy, indicating that risk could be quantified even before birth, with more precise estimation in early years than straight after birth, when model performance was moderate. These prediction models demonstrate that utilising routinely collected healthcare data can form the basis of a risk identification system to strengthen the long-term preventive element of antenatal and early years care by quantifying clustering of future obesity risk in families.

## Supplementary information

**Additional file 1: Table S1** Estimates of the final models for the prediction of outcome of overweight and obesity (≥91st centile) in children aged 4–5 years. **Table S2** Estimates of the final models for the prediction of outcome of overweight and obesity (≥91st centile) in children aged 4–5 years under in Southampton, UK with and without area-level predictors. **Table S3** Predictive parameters for the outcome of overweight and obesity (≥91st centile) in children aged 4–5 years.

## Data Availability

The data owners are University Hospital Southampton NHS Trust, Solent NHS Trust, and Southern Health NHS Foundation Trust. Anonymised data are only available upon request from the PI (NAA) conditional on approval of the appropriate institutional ethics, research governance processes, and data holders.

## Abbreviations

AIC: Akaike information criteria
AUC: Area under the curve
BMI: Body mass index
EPV: Events per variable
GDM: Gestational diabetes
IMD: Index of multiple deprivation
LSOA: Lower layer Super Output Area
MICE: Multiple imputation by chained equations
NCMP: National Child Measurement Programme
NHS: National Health Service
NICE: National Institute for Health and Care Excellence
NPV: Negative predictive value
PPV: Positive predictive value
SD: Standard deviation
SLOPE: Studying Lifecourse Obesity PrEdictors
UHS: University Hospital Southampton
