# Supplementary material for: Predicting the risk of childhood overweight and obesity at 4–5 years using population-level pregnancy and early-life healthcare data
Source: BMC Med. 2020 May 11;18:105. doi: 10.1186/s12916-020-01568-z (PMC7212594; doi:10.1186/s12916-020-01568-z)
Supplement: Supplementary file 1 — Additional file 1: Table S1 Estimates of the final models for the prediction of outcome of overweight and obesity (≥91st centile) in children aged 4–5 years. Table S2 Estimates of the final models for the prediction of outcome of overweight and obesity (≥91st centile) in children aged 4–5 years under in Southampton, UK with and without area-level predictors. Table S3 Predictive parameters for the outcome of overweight and obesity (≥91st centile) in children aged 4–5 years. [file 12916_2020_1568_MOESM1_ESM.docx]

Table S1: Estimates of the final models for the prediction of outcome of overweight and obesity (≥91st centile) in children aged 4-5 years

| Predictors | Booking | | | Birth | | | Early life (~1 year) | | | Early life (~2 years) | | |
| --- | --- | --- | --- | --- | --- | --- | --- | --- | --- | --- | --- | --- |
|  | Coef | (95% CI) | p | Coef | (95% CI) | p | Coef | (95% CI) | p | Coef | (95% CI) | p |
| Intercept | 0.877 | 0.593 to 1.16 |  | 2.215 | 1.348 to 3.082 |  | -5.186 | -6.318 to -4.053 |  | -10.510 | -11.135 to -9.886 |  |
| Maternal age at booking, years | 1.394 | 0.842 to 1.946 | 0.000 | 1.114 | 0.512 to 1.716 | 0.000 | -0.006 | -0.013 to 0.001 | 0.095 |  |  |  |
| Maternal BMI at booking, kg/m^2^ | -7.061 | -7.507 to -6.615 | 0.000 | -6.371 | -6.835 to -5.908 | 0.000 | -6.733 | -7.25 to -6.215 | 0.000 | -6.687 | -7.253 to -6.122 | 0.000 |
| Maternal smoking status at booking |  |  |  |  |  |  |  |  |  |  |  |  |
| Never smoked | Ref |  |  | Ref |  |  | Ref |  |  | Ref |  |  |
| Ex-smoker | 0.099 | 0.02 to 0.178 | 0.014 | 0.080 | -0.003 to 0.163 | 0.059 | 0.047 | -0.041 to 0.135 | 0.298 | 0.044 | -0.05 to 0.138 | 0.361 |
| Current smoker | 0.436 | 0.341 to 0.532 | 0.000 | 0.583 | 0.481 to 0.685 | 0.000 | 0.532 | 0.419 to 0.645 | 0.000 | 0.536 | 0.414 to 0.659 | 0.000 |
| Maternal educational attainment |  |  |  |  |  |  |  |  |  |  |  |  |
| University or above |  |  |  | Ref |  |  | Ref |  |  | Ref |  |  |
| College |  |  |  | 0.088 | -0.01 to 0.186 | 0.077 | 0.130 | 0.028 to 0.233 | 0.013 | 0.116 | 0.006 to 0.225 | 0.038 |
| Secondary or lower |  |  |  | 0.103 | -0.004 to 0.21 | 0.059 | 0.190 | 0.077 to 0.302 | 0.001 | 0.174 | 0.053 to 0.295 | 0.005 |
| Maternal ethnicity |  |  |  |  |  |  |  |  |  |  |  |  |
| White | Ref |  |  | Ref |  |  |  |  |  | Ref |  |  |
| Mixed | 0.020 | -0.304 to 0.343 | 0.904 | 0.105 | -0.238 to 0.447 | 0.550 | 0.098 | -0.25 to 0.446 | 0.580 | 0.019 | -0.355 to 0.394 | 0.919 |
| Asian | 0.274 | 0.121 to 0.428 | 0.000 | 0.444 | 0.283 to 0.605 | 0.000 | 0.589 | 0.424 to 0.754 | 0.000 | 0.402 | 0.223 to 0.581 | 0.000 |
| Black/African/Caribbean | 0.655 | 0.418 to 0.892 | 0.000 | 0.778 | 0.519 to 1.037 | 0.000 | 0.771 | 0.507 to 1.034 | 0.000 | 0.511 | 0.226 to 0.796 | 0.000 |
| Other | 0.084 | -0.267 to 0.434 | 0.640 | 0.124 | -0.237 to 0.484 | 0.501 | 0.235 | -0.153 to 0.624 | 0.235 | -0.073 | -0.487 to 0.341 | 0.729 |
| Maternal intake of folic acid supplements |  |  |  |  |  |  |  |  |  |  |  |  |
| Taking prior to pregnancy | Ref |  |  | Ref |  |  | Ref |  |  | Ref |  |  |
| Started taking once pregnant | 0.094 | 0.013 to 0.175 | 0.023 | 0.120 | 0.037 to 0.203 | 0.005 | 0.156 | 0.067 to 0.245 | 0.001 | 0.155 | 0.058 to 0.252 | 0.002 |
| Not taking supplement | 0.053 | -0.072 to 0.178 | 0.402 | 0.084 | -0.044 to 0.213 | 0.198 | 0.160 | 0.023 to 0.296 | 0.022 | 0.159 | 0.002 to 0.317 | 0.047 |
| Maternal first language English |  |  |  |  |  |  |  |  |  |  |  |  |
| No | Ref |  |  | Ref |  |  |  |  |  |  |  |  |
| Yes | -0.319 | -0.515 to -0.122 | 0.001 | -0.285 | -0.496 to -0.074 | 0.008 |  |  |  |  |  |  |
| Partnership status at booking |  |  |  |  |  |  |  |  |  |  |  |  |
| Partnered | Ref |  |  | Ref |  |  | Ref |  |  | Ref |  |  |
| Single | 0.182 | 0.067 to 0.297 | 0.002 | 0.193 | 0.075 to 0.31 | 0.001 | 0.196 | 0.066 to 0.327 | 0.003 | 0.176 | 0.033 to 0.319 | 0.016 |
| Parity at booking |  |  |  |  |  |  |  |  |  |  |  |  |
| 0 | Ref |  |  | Ref |  |  |  |  |  |  |  |  |
| 1 | 0.017 | -0.062 to 0.095 | 0.678 | -0.108 | -0.186 to -0.029 | 0.007 |  |  |  |  |  |  |
| 2 | 0.093 | -0.014 to 0.2 | 0.088 | -0.057 | -0.169 to 0.055 | 0.318 |  |  |  |  |  |  |
| 3 | 0.173 | 0.037 to 0.308 | 0.012 | 0.019 | -0.125 to 0.162 | 0.799 |  |  |  |  |  |  |
| Birthweight, kg |  |  |  | 0.107 | 0.097 to 0.117 | 0.000 | 0.129 | 0.031 to 0.226 | 0.010 | -0.114 | -0.196 to -0.032 | 0.007 |
| Gestational age at birth, days |  |  |  | -0.011 | -0.014 to -0.008 | 0.000 | -0.008 | -0.012 to -0.004 | 0.000 |  |  |  |
| Child sex |  |  |  |  |  |  |  |  |  |  |  |  |
| Male |  |  |  |  |  |  | Ref |  |  | Ref |  |  |
| Female |  |  |  |  |  |  | 0.426 | 0.343 to 0.509 | 0.000 | 0.366 | 0.283 to 0.45 | 0.000 |
| Child weight, kg |  |  |  |  |  |  | 0.753 | 0.694 to 0.813 | 0.000 | 0.825 | 0.78 to 0.869 | 0.000 |

Table S2: Estimates of the final birth models for the prediction of outcome of overweight and obesity (≥91^st^ centile) in children aged 4-5 years in the Southampton area, with and without area-level predictors* (n=13878)

| Predictors | Birth, no area | | | Birth and IMD, no education | | | Birth and all area | | |
| --- | --- | --- | --- | --- | --- | --- | --- | --- | --- |
|  | Coef | (95% CI) | p | Coef | (95% CI) | p | Coef | (95% CI) | p |
| Intercept | -1.919 | -2.207 to -1.632 |  | -1.713 | -1.981 to -1.445 |  | -1.840 | -2.173 to -1.51 |  |
| Maternal age at booking, years | -0.010 | -0.020 to -0.001 | 0.036 | -0.011 | -0.020 to -0.001 | 0.027 | -0.009 | -0.018 to 0.00 | 0.079 |
| Maternal BMI at booking, kg/m^2^ | 0.663 | 0.555 to 0.771 | 0.000 | -5.942 | -6.597 to -5.286 | 0.000 | -5.890 | -6.546 to -5.23 | 0.000 |
| Maternal smoking status at booking |  |  |  |  |  |  |  |  |  |
| Never smoked | Ref |  |  |  |  |  |  |  |  |
| Ex-smoker | 0.135 | 0.011 to 0.259 | 0.033 | 0.143 | 0.019 to 0.266 | 0.024 | 0.131 | 0.007 to 0.26 | 0.039 |
| Current smoker | 0.595 | 0.453 to 0.737 | 0.000 | 0.601 | 0.460 to 0.742 | 0.000 | 0.579 | 0.437 to 0.72 | 0.000 |
| Maternal educational attainment |  |  |  |  |  |  |  |  |  |
| University or above | Ref |  |  |  |  |  | Ref |  |  |
| College | 0.136 | -0.016 to 0.287 | 0.079 |  |  |  | 0.113 | -0.039 to 0.27 | 0.144 |
| Secondary or lower | 0.184 | 0.024 to 0.344 | 0.025 |  |  |  | 0.162 | 0.001 to 0.32 | 0.048 |
| Maternal employment |  |  |  |  |  |  |  |  |  |
| Employed | Ref |  |  | Ref |  |  | Ref |  |  |
| Unemployed and seeking work | -0.149 | -0.262 to -0.036 | 0.010 | -0.143 | -0.255 to -0.031 | 0.012 | -0.158 | -0.271 to -0.04 | 0.006 |
| Full/part-time students/trainees | -0.066 | -0.365 to 0.233 | 0.666 | 0.091 | -0.390 to 0.209 | 0.552 | -0.071 | -0.370 to 0.23 | 0.643 |
| Maternal ethnicity |  |  |  |  |  |  |  |  |  |
| White | Ref |  |  | Ref |  |  | Ref |  |  |
| Mixed | 0.539 | 0.159 to 0.919 | 0.005 | 0.531 | 0.151 to 0.910 | 0.006 | 0.545 | 0.166 to 0.92 | 0.005 |
| Asian | 0.521 | 0.335 to 0.707 | 0.000 | 0.496 | 0.310 to 0.681 | 0.000 | 0.523 | 0.337 to 0.71 | 0.000 |
| Black/African/Caribbean | 0.790 | 0.482 to 1.098 | 0.000 | 0.765 | 0.458 to 1.071 | 0.000 | 0.772 | 0.465 to 1.08 | 0.000 |
| Other | 0.136 | -0.318 to 0.590 | 0.558 | 0.104 | -0.349 to 0.557 | 0.651 | 0.124 | -0.332 to 0.58 | 0.595 |
| Maternal intake of folic acid supplements |  |  |  |  |  |  |  |  |  |
| Taking prior to pregnancy | Ref |  |  | Ref |  |  | Ref |  |  |
| Started taking once pregnant | 0.128 | 0.000 to 0.255 | 0.050 | 0.131 | 0.004 to 0.259 | 0.044 | 0.116 | -0.012 to 0.24 | 0.075 |
| Not taking supplement | 0.159 | -0.021 to 0.339 | 0.083 | 0.168 | -0.011 to 0.347 | 0.065 | 0.147 | -0.033 to 0.33 | 0.109 |
| Maternal first language English |  |  |  |  |  |  |  |  |  |
| No | Ref |  |  | Ref |  |  | Ref |  |  |
| Yes | -0.248 | -0.478 to -0.019 | 0.034 | -0.272 | -0.499 to -0.045 | 0.019 | -0.244 | -0.474 to -0.01 | 0.037 |
| Lone parent at booking |  |  |  |  |  |  |  |  |  |
| No | Ref |  |  | Ref |  |  | Ref |  |  |
| Yes | 0.256 | 0.104 to 0.407 | 0.001 | 0.252 | 0.101 to 0.404 | 0.001 | 0.247 | 0.095 to 0.40 | 0.001 |
| Birthweight, kg | 0.663 | 0.555 to 0.771 | 0.000 | 0.666 | 0.558 to 0.774 | 0.000 |  |  |  |
| Gestational age at birth, days | 0.010 | -0.015 to -0.005 | 0.000 | -0.010 | -0.015 to -0.006 | 0.000 | 0.010 | -0.015 to -0.01 | 0.000 |
| Household median price quintile (England) |  |  |  |  |  |  |  |  |  |
| Cheapest quintile |  |  |  |  |  |  | Ref |  |  |
| 2nd quintile |  |  |  |  |  |  | 0.031 | -0.146 to 0.21 | 0.735 |
| 3rd quintile |  |  |  |  |  |  | -0.118 | -0.306 to 0.07 | 0.216 |
| 4th quintile |  |  |  |  |  |  | -0.136 | -0.378 to 0.11 | 0.270 |
| Most expensive quintile |  |  |  |  |  |  | -0.386 | -0.753 to -0.02 | 0.039 |
| IMD quintile Hampshire |  |  |  |  |  |  |  |  |  |
| Most deprived |  |  |  | Ref |  |  |  |  |  |
| 2nd quintile |  |  |  | -0.096 | -0.217 to 0.024 | 0.117 |  |  |  |
| 3rd quintile |  |  |  | -0.161 | -0.332 to 0.009 | 0.064 |  |  |  |
| 4th quintile |  |  |  | -0.175 | -0.394 to 0.045 | 0.119 |  |  |  |
| Least deprived |  |  |  | -0.256 | -0.671 to 0.159 | 0.227 |  |  |  |
| **Transformations:** |  |  |  |  |  |  |  |  |  |
| Maternal age at booking | Maternal age at booking -27.51463246 | | | Maternal age at booking -27.51463246 | | | Maternal age at booking -27.51463246 | | |
| Maternal BMI at booking | (Maternal BMI/10)^-1 -.3894709131 | | | (Maternal BMI/10)^-1 -.3894709131 | | | (Maternal BMI/10)^-1 -.3894709131 | | |
| Birthweight | Birthweight - 3.377177356 | | | Birthweight - 3.377177356 | | | Birthweight - 3.377177356 | | |
| Gestational age at birth (days) | Gestational age at birth - 278.4070973 | | | Gestational age at birth - 278.4070973 | | | Gestational age at birth - 278.4070973 | | |
| AUC | 0.6788 | | | 0.6794 | | | 0.6807 | | |

*Area-level predictors considered for inclusion in the model include Index of Multiple Deprivation (IMD), household median price quintile, measures of air pollution (PM2.5, PM10, NOx), Income Support claimant rate, social renting households, supermarket density, unhealthy food index, greenspace, spaces for social interaction and walkability

Table S3: Predictive parameters for the outcome of overweight and obesity (≥91st centile) in children aged 4-5 years

| Cut-point | % at or above cut-point | Sensitivity | Specificity | Positive predictive value (PPV) | Negative predictive value (NPV) |
| --- | --- | --- | --- | --- | --- |
| Booking |  |  |  |  |  |
| ≥10.0 | 69.0 | 84.7  83.6 to 85.9 | 33.7  33.1 to 34.3 | 18.2  17.7 to 18.7 | 92.7  92.1 to 93.2 |
| ≥15.0 | 39.3 | 59.5  58.0 to 61.0 | 64.2  63.6 to 64.8 | 22.5  21.7 to 23.2 | 90.1  89.7 to 90.5 |
| ≥20.0 | 21.2 | 37.1  35.6 to 38.5 | 81.6  81.1 to 82.1 | 25.9  24.8 to 27.0 | 88.2  87.7 to 88.6 |
| ≥25.0 | 11.1 | 22.2  20.9 to 23.4 | 90.8  90.5 to 91.1 | 29.6  28.1 to 31.3 | 87.0  86.6 to 87.4 |
| ≥30.0 | 5.1 | 12.3  11.3 to 13.3 | 96.1  95.8 to 96.3 | 35.3  32.9 to 42.7 | 86.3  85.9 to 86.7 |
| Birth |  |  |  |  |  |
| ≥10.0 | 64.2 | 83.9  82.7 to 84.9 | 39.3  38.6 to 39.9 | 19.4  18.8 to 20.0 | 93.3  92.8 to 93.8 |
| ≥15.0 | 38.1 | 60.8  59.4 to 62.3 | 65.9  65.3 to 66.4 | 23.7  22.9 to 24.5 | 90.6  90.2 to 91.0 |
| ≥20.0 | 21.8 | 41.4  39.9 to 42.9 | 81.6  81.1 to 82.1 | 28.1  29.9 to 29.2 | 88.9  88.5 to 89.3 |
| ≥25.0 | 12.6 | 27.7  26.3 to 29.0 | 90.0  89.6 to 90.4 | 32.5  31.9 to 34.1 | 87.7  87.3 to 88.1 |
| ≥30.0 | 7.0 | 16.9  15.8 to 18.1 | 94.7  94.4 to 95.0 | 35.6  33.6 to 37.8 | 86.7  86.3 to 87.1 |
| Early life (~1 year) |  |  |  |  |  |
| ≥10.0 | 50.9 | 83.6  82.5 to 84.7 | 54.8  54.1 to 55.4 | 24.3  23.7 to 25.0 | 95.0  94.7 to 95.4 |
| ≥15.0 | 34.7 | 69.6  68.2 to 70.9 | 71.3  70.8 to 71.9 | 29.7  28.8 to 30.6 | 93.1  92.7 to 93.4 |
| ≥20.0 | 24.3 | 56.9  55.4 to 58.4 | 81.4  80.9 to 81.9 | 34.2  33.6 to 35.9 | 91.6  91.2 to 91.9 |
| ≥25.0 | 17.3 | 46.0  44.5 to 47.5 | 87.7  87.3 to 88.1 | 39.4  38.9 to 40.8 | 90.3  89.9 to 90.7 |
| ≥30.0 | 12.5 | 36.8  35.4 to 38.3 | 91.7  91.4 to 92.1 | 43.7  42.1 to 45.3 | 89.3  88.9 to 89.7 |
| Early life (~2 years) |  |  |  |  |  |
| ≥10.0 | 43.5 | 84.6  83.5 to 85.6 | 63.7  63.1 to 64.3 | 28.9  28.1 to 29.7 | 96.0  95.6 to 96.2 |
| ≥15.0 | 31.8 | 74.8  73.5 to 76.1 | 75.7  75.2 to 76.2 | 34.9  33.9 to 35.9 | 94.5  94.2 to 94.8 |
| ≥20.0 | 24.1 | 65.5  64.0 to 66.9 | 83.1  82.6 to 83.6 | 40.3  39.1 to 41.4 | 93.3  92.9 to 93.6 |
| ≥25.0 | 18.8 | 57.2  55.7 to 58.6 | 87.9  87.5 to 88.3 | 45.2  43.9 to 46.5 | 92.2  91.8 to 92.5 |
| ≥30.0 | 14.8 | 59.7  58.2 to 51.2 | 91.3  90.9 to 91.6 | 49.9  48.4 to 50.7 | 91.2  90.9 to 91.6 |
